# Vintage electronics for trusted radiation measurements and verified dismantlement of nuclear weapons

**DOI:** 10.1371/journal.pone.0224149

**Published:** 2019-10-30

**Authors:** Moritz Kütt, Alexander Glaser

**Affiliations:** 1 Institute for Peace Research and Security at the University of Hamburg, Germany; 2 Program on Science and Global Security, Princeton University, Princeton, NJ, United States of America; Wuhan University, CHINA

## Abstract

Information barriers are trusted measurement systems to confirm the authenticity of nuclear warheads based on their radiation signatures. Traditional inspection systems rely on complex electronics both for data acquisition and processing. Several research efforts have produced prototype systems, but it has proven difficult to demonstrate that hidden switches and side channels do not exist. After almost thirty years of research and development, no viable and widely accepted system has emerged. We pursue a fundamentally different approach: Our prototype of an inspection system uses vintage hardware built around a 6502 processor. The processor uses 8-micron technology and has only about 4,200 transistors. Vintage electronics may have a number of important advantages for applications where two parties need to simultaneously establish trust in the hardware used. CPUs designed in the distant past, at a time when their use for sensitive measurements was never envisioned, drastically reduce concerns that the other party implemented backdoors or hidden switches on the hardware level. We demonstrate the performance of a prototype system using an Apple IIe and a custom-made open-source data-processing board connected to a standard sodium-iodide radiation detector for low-resolution gamma spectroscopy. Data processing and analysis is exclusively done on the Apple IIe hardware. We show that subtle differences in radiation signatures can be detected in 2–3 minutes based on the result of a simple chi-squared test. Vintage electronics may therefore offer a new path toward fieldable, trusted information barriers.

## Introduction

The design of nuclear weapons remains one of the most closely held secrets today. In particular, the amounts and configuration of nuclear materials (namely, plutonium and highly enriched uranium) in a specified warhead type are considered highly sensitive information by all nuclear weapon states. At the same time, future arms control agreements may place limits on the number of nuclear weapons in the arsenals, including those in storage, and could require inspections to confirm the authenticity of these weapons prior to dismantlement. All nuclear weapons contain radioactive materials, and it is possible to use gamma and neutron radiation signatures for verification purposes [1, 2]. In general, these measurements would reveal design information and could be used to reverse engineer or otherwise characterize the weapon. To avoid this situation but still enable confirmation measurements, the use of dedicated inspection systems with information barriers has been proposed and pursued since the early 1990s [3–6].

Information barriers are systems, typically part of or connected to a standard radiation detector, that acquire and process sensitive data and display the results of an analysis in a simple, pass/fail manner. Both host and inspector need to simultaneously trust the device, creating two separate functional requirements: The host party must be confident that there are no side channels that could leak restricted information, either deliberately or accidentally, for example during a malfunction of the device. At the same time, the inspector party must be confident that the device presents an output based on true measurement results, which requires, in particular, that the device does not contain any hidden switches or other cheating mechanisms that could be used by the host to manipulate the measurements or the results in an attempt to affect the outcome of the inspection. These processes are called certification and authentication of the equipment [7].

Two measurement approaches for warhead confirmation measurements have been proposed. For the so-called attribute approach, the inspection system determines a number of properties and accepts an item as valid if the results meet specific criteria, such as the presence and a minimum amount of a particular material, for example, plutonium [8]. Properties, thresholds, and ranges have to be negotiated prior to deployment and use. In contrast, inspection systems based on the template-matching approach compare measured data to a reference dataset (template) that has been recorded from a trusted reference item, which can be obtained, for example, by randomly selecting a warhead directly from its delivery system under inspector supervision. A fundamental disadvantage of these systems is the need to acquire and then securely store the template, which itself would contain sensitive information [9].

After thirty years of R&D, no viable inspection system with information barrier has been successfully demonstrated. For this to be the case, a host would not object to measurements on classified items, while an inspector would have high confidence in the outcome of such a measurement, which could require direct access to the equipment after the inspection is complete. These criteria have never been met simultaneously. Fundamentally, this dilemma is rooted in a lack of trust in the electronics that process the data and protect the sensitive information.

In an attempt to facilitate confidence in verification systems and to resolve the certification/authentication dilemma, here, we propose the use of vintage electronics from the 1970s, i.e., from a time when integrated circuits first became widely available. Establishing trust in such hardware should be easier for at least two reasons. First, old hardware is significantly less capable than modern electronics, by factors of thousands or even millions. The limits on processing power makes the implementation of exploitable software backdoors or hidden switches extremely difficult. Second, it is implausible that microprocessors produced more than forty years ago would have been manufactured with suitable hardware vulnerabilities, introduced accidentally or on purpose at the time, that remain undetected to this day but could be used to defeat information barriers whose software and hardware architectures have not been specified yet.

Our new prototype information barrier (IBX II) measures gamma radiation and follows the template approach. While remaining as simple as possible, it needs sufficient processing capabilities to carry out the following functions: digitize pulses from a scintillation detector with photomultiplier; create a histogram of different pulse heights, i.e., serve as a multichannel analyzer; calibrate the recorded spectrum; resample the spectrum into bins combining several channels; and check two sets of bins for similarity using simple statistical tests.

## Review of existing systems

Most previous information barrier projects discussed simplicity as an important confidence-building measure. With a few exceptions, however, they failed to live up to this goal. The following gives a brief overview of relevant systems, with a particular focus on their hardware choices and data-processing capabilities. Table 1 shows the main specifications of selected systems.

**Table 1:** Data processing parts of different information barrier prototypes.

System	Processor	Architecture	Clock	OS
CIVET	Intel 186, FPGA	16 Bit	6–25MHz	custom
AMS/IB	Intel 386SX	32 Bit	25 MHz	ROM-DOS 6.22
	Intel 486DX	32 Bit	66 MHz	ROM-DOS 6.22 / Windows
NG-AMS	x86 (Data acquisition)	32 Bit	n/a	Windows XP
	8051 (Barrier)	8 Bit	12 MHz	custom
TRIS	586	32 Bit	n/a	ROM-DOS 6.22
NG-TRIS	AMD 586, FPGA	32 Bit	133 MHz	ROM-DOS 6.22
UKNI	ATmega 2560	8 Bit	16 MHz	custom
IBX	Xilinx Zynq-7010	32 Bit	866 MHz	Linux
IBX II	MOS 6502	8 Bit	1 MHz	Apple IIe ROM

Under development at Brookhaven National Laboratory since the late 1980s, the “Controlled Intrusiveness Verification Technology” (CIVET) system was one of the earliest information barriers. CIVET was based on the attribute approach and used a high-purity germanium (HPGe) detector with commercial hardware for analog-to-digital conversion. The system ran custom software without a multipurpose operating system [3, 10]. Additional information barriers based on the attribute approach were later pursued as part of the Trilateral Initiative between Russia, the United States, and the International Atomic Energy Agency (IAEA). To support this initiative, U.S. researchers developed the “Attribute Measurement System with Information Barrier” (AMS/IB), which measured six different attributes using a HPGe detector and a neutron multiplicity measurement system. Four computers acquired the data from several detectors, a fifth combined the data from the others and enabled user interaction [11, 12]. The “Next Generation Attribute Measurement System” (NG-AMS) followed a similar measurement approach, but used a single computer running *Windows XP Embedded* and other commercial software [13, 14]. The information barrier itself was built in a very simple way, using an 8-bit microcontroller (*Intel 8051* architecture) and custom software. A new iteration of this system (“Third Generation Attribute Measurement System,” 3G-AMS) has been proposed, but no prototype was ever built [15]. The UK-Norway Initiative, launched in 2007, is the most recent development effort of an information barrier based on the attribute approach. It was the first effort to develop an information barrier involving a nuclear weapon state and a non-nuclear weapon state. The project resulted in two different prototypes. The first device was able to confirm the presence of cobalt-60 (as a substitute for radioactive materials expected in a nuclear weapon), while the second device was designed to determine the ratio between plutonium-239 and plutonium-240 in a sample. This last prototype consists of several modules, all custom-made but relying on commercially available parts. A modern 8-bit microcontroller, the *Atmel ATmega2560*, is used for data processing. The software is open source and, along with the hardware schematics, is freely available on the project website [16, 17].

Along with attribute-based systems, other efforts pursued the development of information barriers based on the template-matching method. In general, template-based concepts offer the possibility of using simple, low-resolution detectors and much shorter measurement times (on the order of a few minutes versus several hours). The most prominent information barrier based on the template approach is the “Trusted Radiation Inspection System” (TRIS) developed by Sandia National Laboratories. TRIS acquires gamma spectra using a standard sodium-iodide (NaI) detector. Data is processed using two separate computers (red side, black side) in a tamper-proof enclosure running ROM-DOS 6.22 and custom software. Early versions used a commercial multichannel analyzer separate from the enclosure, later versions used a custom-made analyzer based on a field-programmable gate array (FPGA) within the enclosure [18–20]. Following the general concepts that are the basis of TRIS, in 2015, our team built a first information barrier for prototyping purposes, the Information Barrier Experimental (IBX). The device uses a very fast digitizing system, the Red Pitaya board [21]. The release version of the board features a 125 MS/s analog-to-digital converter, an FPGA for data processing, and an ARM Cortex A9 microprocessor. Although Red Pitaya software and FPGA modules are open source, the processor, the FPGA bitstream and the board circuit are proprietary. Data acquired by the detector are processed on the FPGA and analyzed using Python scripts running under Linux on the Red Pitaya. During the design stage of the IBX, we made a conscious effort to minimize the number of components on the analog front-end of the device. In addition to the Red Pitaya board, only a high-voltage supply for the photomultiplier is needed, which made possible a particularly simple, transparent, and affordable design [22].

In summary, and despite efforts to develop simple and modular devices, all currently existing information barriers rely on relatively complex hardware or software. Often, the data processing system schematics are proprietary. Several systems include FPGAs, or make use of external electronics, for example, commercial multichannel analyzers. Such systems typically have additional microcontrollers and use internal, proprietary firmware. On the software side, systems either use commercial operating systems and data processing tools or custom code. Hardware schematics and software of the UKNI information barrier and the IBX are considered open source and most has been made publicly available. In the new project presented here, we build upon these experiences, but use a radically different approach to simplify the hardware itself in order to establish trust in the measurement process and the inspection results.

## Apple IIe (MOS 6502) information barrier

To demonstrate that vintage computers are in fact capable of carrying out the required computations, we developed the Information Barrier Experimental II or IBX II. The device is based on an Apple IIe and a MOS 6502 microprocessor, both manufactured in the early 1980s. With its eight expansion slots, the Apple IIe is an excellent prototyping platform. It is extremely versatile and well documented, both in terms of the hardware specifications and the software implemented on the Apple ROM.

**Fig 1:**
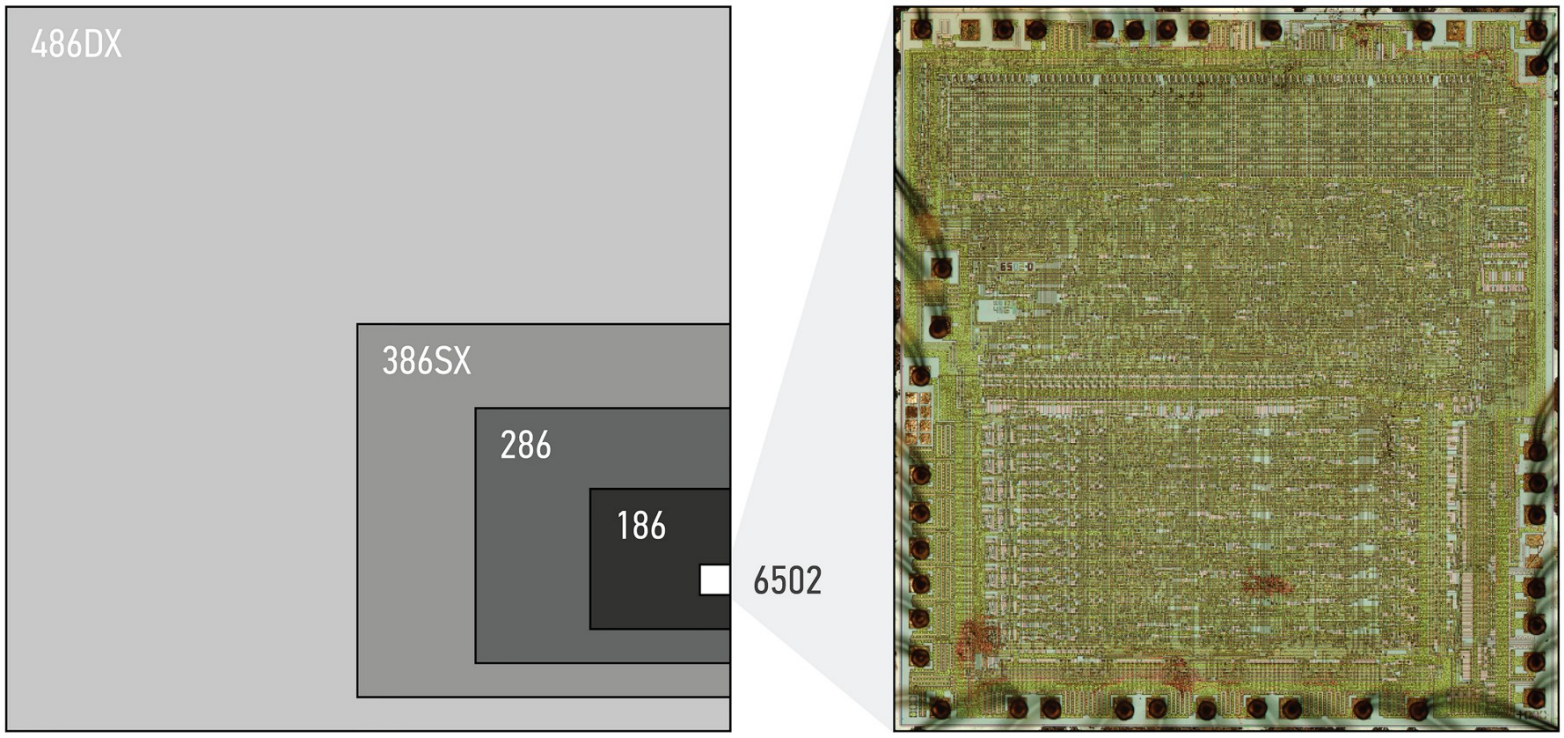
Comparing die sizes and transistor counts. Relative die sizes of different processors had they been produced with the same manufacturing process as the 6502 shown on the right. Areas scale roughly with the number of transistors on the chip. The size of the 6502 die is 3.9 × 4.3 mm^2^. *High-resolution photograph of the 6502 die: visual6502.org*.

The MOS 6502 microprocessor used in the Apple IIe and other contemporary home computers, game consoles, and arcade machines was developed in the 1970s. Even then, it was simpler than other processors of the era. The 6502 uses only 4,237 transistors, of which 1,019 act as pull-up resistors [23], compared to millions of transistors used in processors from the early 1990s and billions of transistors used in today’s processors (Fig 1). Production of the 6502 continues to this date, and the current manufacturer (*Western Design Center, Inc*.) estimates that total global production numbers could have reached up to ten billion units. Although original 6502 design information is not available, various reverse-engineering projects collected such information. Numerous software emulators exist and allow the execution of 6502 binaries, thus replicating the chip at the instruction level [24]. Implementations in hardware description languages can be transferred to FPGAs for chip replication at the level of logic gates. The *Visual 6502* project constructed a complete circuit schematic using high-resolution die photography [25]. Based on this schematic, the *Monster 6502* project produced a transistor-level replica in hardware [23], and other transistor-level replications exist for the use on FPGAs [26]. No other microprocessor may be better understood at the transistor level than the 6502 today.

**Fig 2:**
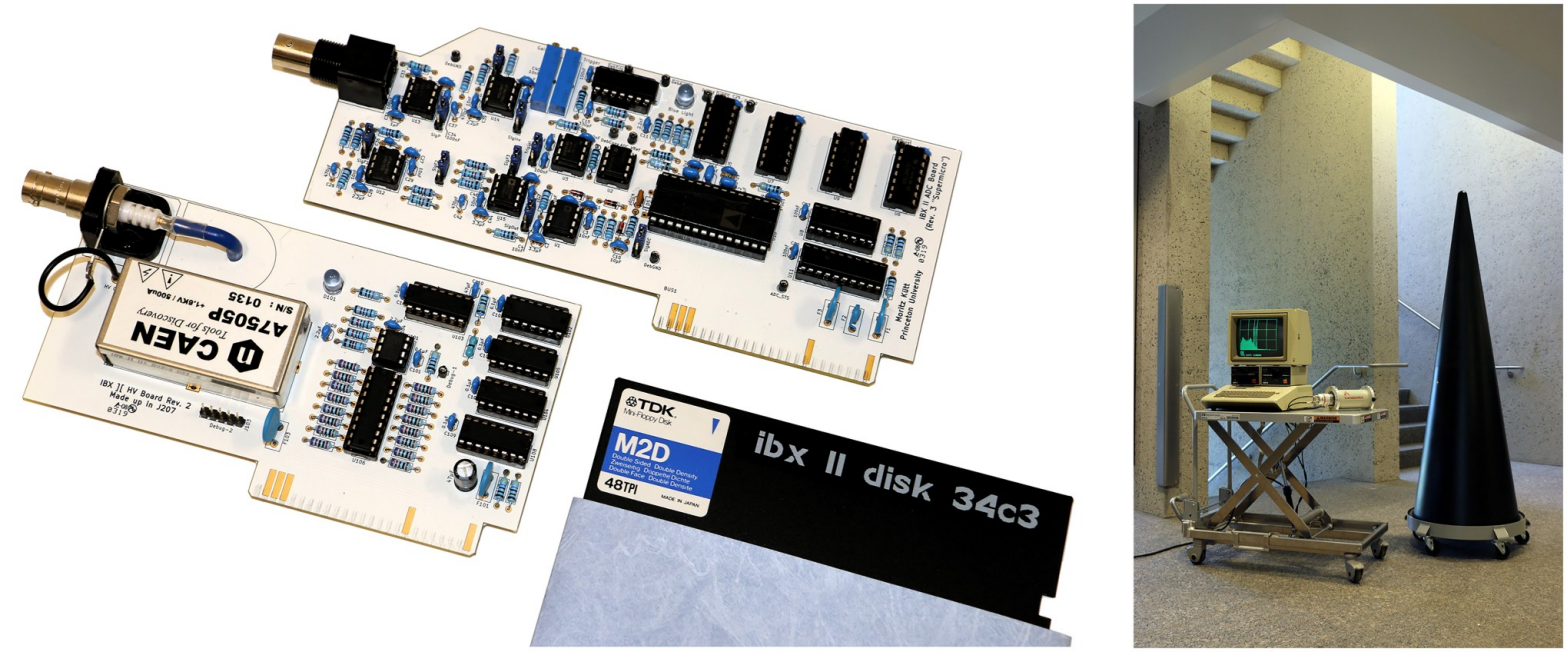
IBX II extension cards (left) and measurement setup with mockup warhead (right). The first card provides high voltage for the photomultiplier, the second card acquires and processes the voltage pulses from the detector. Calibration sources can be emplaced in the mockup warhead to simulate inspection conditions. In practice, warheads or other treaty-limited items may be presented in containerized form.

Our prototype information barrier (Fig 2, right) follows the template approach based on gamma spectroscopy with a standard low-resolution sodium-iodide scintillation detector and photomultiplier (Canberra/Mirion Technologies Model 802 [27]). A gamma spectrum is generated by counting the number of gamma rays detected for each energy bin in a defined range, typically between 0–3000 keV. These spectra are characteristic for isotopic composition, mass, and geometry of the emitting object.

In order to use the detector with an Apple IIe, we designed and built two dedicated extension cards for the machine (Fig 2, left); the schematics of these cards are open source and freely available [28]. The first card contains a circuit to provide the high voltage for the photomultiplier. It uses a compact power supply module (CAEN A7505P), mainly for safety reasons, but it would be straightforward to replace this module with discrete parts for additional transparency of the hardware. The second card digitizes incoming events. It consists of an analog signal processing section to amplify and condition the signal coming from the photomultiplier. The amplification gain is adjusted so that voltage pulses of detected events have a voltage of about 10 V for 3-MeV gammas. A custom peak-detect-and-hold circuit holds peak voltage until digitization is complete. An analog comparator with adjustable trigger level is used to detect peaks and trigger subsequent conversion by a 12-bit AD1674 analog-to-digital converter (ADC). The ADC has an 8-bit bus interface. After conversion is complete, only the 8 most significant bits are read by the 6502, resulting in 256 available channels. A second read operation resets the trigger circuit to allow for new events to be detected. The process of peak detection, data conversion, and transfer of the data to the memory of the Apple IIe takes about 50 *μ*s for a single event. During that time, no other signal can be detected. Hence, the theoretically achievable count rate therefore approaches 20,000 counts per second. Because the individual events are randomly distributed in time, significant pile-up effects would occur at such count rates. At count rates of about 2000 counts per second, pile-up will occur only in a fraction of 9.5% of all counts. Such a count rate is a typical value expected for treaty verification measurements at a standoff distance of one meter. For the measurements with IBX II, the actual criteria to end a single measurement is the total number of counts (currently 262,144 total counts). At 2000 counts per second, IBX II obtains statistically robust data in 2-3 minutes. The statistical uncertainty for counts in an average channel in this case is 3.2%/2.7%. For the current prototype, results are displayed on a monitor, but a fieldable device would likely use a small number of LEDs to indicate system status and outcome of the inspection.

## Measurement approach & algorithm

The control software for the two cards is written in 6502 assembler language. The code provides a simple user interface to control basic tasks: toggle high voltage; acquire new template; load existing template; inspect item; and display results. The details of the envisioned inspection protocol have been described elsewhere [29].

The system autocalibrates “on the fly” using thoriated welding rods, which are wrapped around the detector crystal and emit a characteristic 2.614 MeV peak. A 6502-based algorithm searches for this peak and scales the data such that its energy corresponds to Channel 226. Relying only on integer multiplication and division, peak finding and scaling is performed internally with 16-bit resolution. The center of the peak is identified using a binary search to look for a fixed-size energy window where the average channel of the events in this window matches the center channel of the selected window. To correct for non-linear effects in the detector response and front-end pulse processing, a lookup table is used to convert channel numbers to gamma energies. This table has to be generated at least once (upon first assembly and testing of the system) and is then used for all future measurements. It is worth pointing out that exact energy calibration and linearity of detector response are not critical for the performance of the device as long as measurement results are reproducible.

For a typical spectrum, 262,144 (2^18^) events are recorded. In practice, we allocate two bytes (i.e., up to 65,535 counts) per channel for a total of 512 bytes of memory. The IBX II discrimination algorithm is inspired by the method used for TRIS, which compares the acquired spectrum to a relatively simple template with fewer than twenty bins. Similarly, the IBX II algorithm merges the data into twelve broad-energy bins, combining data from multiple channels into these bins. The distribution of counts for the template is then compared to the distribution of the measured item using a simple *χ*^2^ statistical test, where measurement bins *M*_*i*_ are compared to the result for the template with data in bins *T*_*i*_.

$$\begin{array}{c} \chi^{2}=\sum_{i=1}^{12}\frac{{\left(M_{i}-T_{i}\right)}^{2}}{T_{i}} \end{array}$$ (1)

Acceptable *χ*^2^ values depend on expected differences among valid objects (for example, manufacturing tolerances and age relevant for a particular weapon type) and would have to be negotiated among inspecting party and host prior to inspections. For use as an inspection system in a true arms-control setting, the number of bins, their widths, and their weights could be modified based on the recommendations of the negotiating parties; here, we are primarily interested in the proof-of-concept.

The *χ*^2^ is used to reliably compare distributions with total count rates in the range of hundreds of counts. However, IBX II records significantly more events to ensure that the position of the calibration peak can be precisely determined. In the 450 measurements described below, the average height of the calibration peak was approx. 130 counts in the central channel.

To automatically record a large number of spectra for testing and evaluation purposes, the prototype IBX II includes a routine that can store spectra on a 5¼-inch floppy disk, which is also used to load the main program during the boot process.

## Results

**Fig 3:**
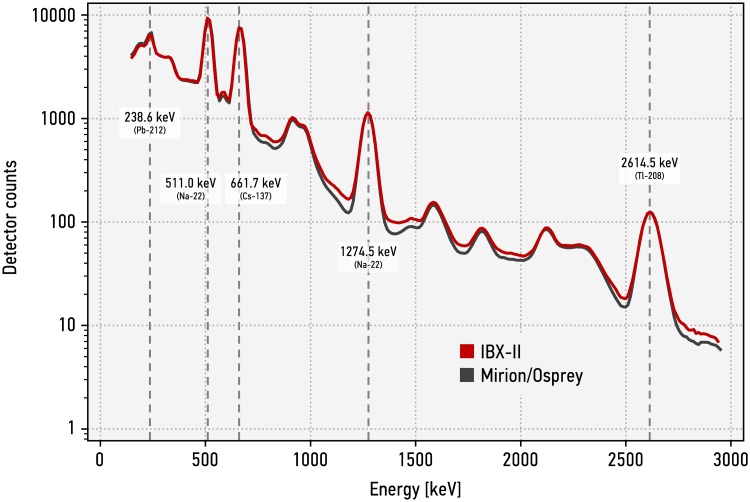
IBX II (256-channel) gamma spectrum compared to a spectrum acquired with a modern detector system. A few prominent gamma energies are highlighted. Shown spectra are averaged data from 150 standard measurements with 2^18^ events each.

Fig 3 shows a sample spectrum acquired with the IBX II compared to a spectrum acquired with a modern detector system (Canberra/Mirion Osprey) for the same source-detector configuration. The collected spectral data for all measurements discussed here are published via a GitHub repository (http://github.com/sgs-lab/ibxII-measurements). The measurement used a sodium-22 and a caesium-137 source (Spectrum Techniques) with a nominal activity of 37 kBq each at time of production (11/2014). The source position was 35 mm away from the surface of the sodium-iodide crystal, vertically at the center line of the detector, with the sodium-22 source closer to the detector. For the calibration, twelve 1-inch pieces of 4 mm thick welding rods surrounded the scintillator crystal in a 3D-printed holder, around the front end of the detector, i.e. away from the photomultiplier. The measurements with IBX II set the photomultiplier voltage to 1000 V. For the measurements using the Canberra/Mirion Osprey, Fine and Coarse Gain of the Osprey were set to 1, the photomultiplier voltage was set to 870 V. The recorded 4096 channels were condensed into 256 channels by a Python script to allow for better comparison.

The performance of both systems is essentially identical. From these data, the detector resolution (defined as the full-width at half-maximum versus the centroid energy of the peak) of the IBX II can be estimated to 6.8% at 661.7 keV (caesium-137) and to 3.6% at 2614.5 keV (thallium-208). These numbers are limited by the fundamental phenomena in the sodium-iodide crystal. IBX II does not acquire gamma energies below 150 keV because these are de-facto irrelevant for inspection applications as they could be shielded relatively easily by intervening materials (for example, by the container in which the inspected item is presented) or by a host trying to manipulate the outcome of the inspection. Low-energy gammas therefore do not provide a robust signature for inspection applications.

**Fig 4:**
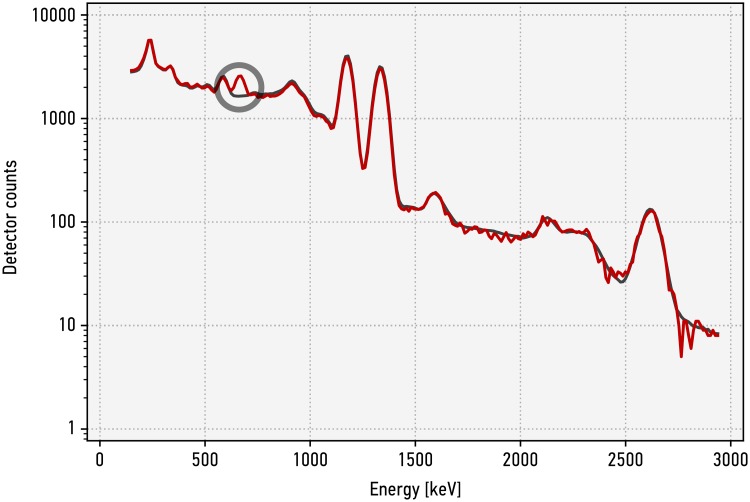
IBX II gamma spectra of an invalid item compared to the template. The template values are generated from the average result of 150 measurements on the reference item, represented by a cobalt-60 source. The invalid item is the same cobalt-60 source with a weak caesium-137 contribution (661.7 keV), highlighted in the plot. Shown are the template and a single measurement with 2^18^ total events.

**Fig 5:**
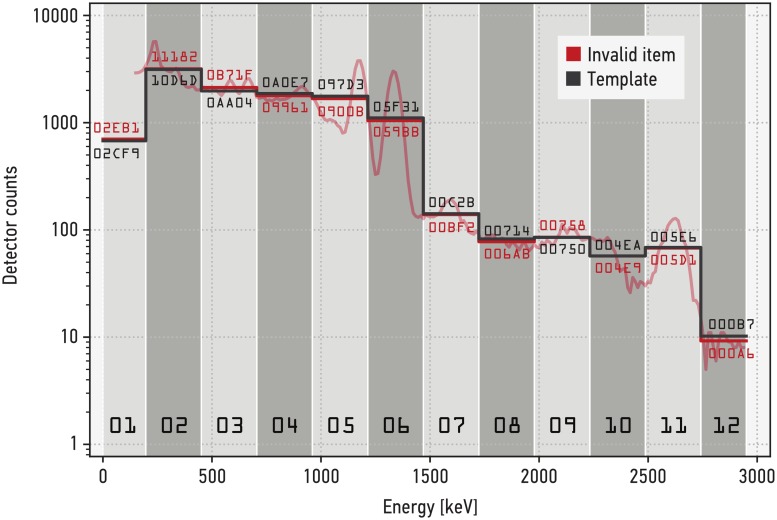
Count rates in IBX II broad-energy bins. The template values (gray line) are used for the *χ*^2^ test in all future inspections. For reference purposes, the spectrum and the corresponding bin values from a single measurement of an invalid item (cobalt-60 source with a weak caesium-137 contribution) are shown (red line). Bin values for the invalid item and the template are in hexadecimal format.

For the following measurements we use two different source configurations, one representing the reference item (used to generate the template) and one representing an invalid candidate item with a small but noticeable spectral difference. The reference item configuration uses a cobalt-60 source, while the invalid item has an additional (weak) cesium-137 contribution. The cobalt-60 source consisted of two Spectrum Techniques cobalt-60 sources with a nominal activity of 37 kBq each at the time of production (10/2014). The source position was 35 mm away from the surface of the sodium-iodide crystal, vertically at the center line of the detector. Thorium welding rods were placed as described above. For the invalid item, a caesium-137 source (described above) was added in a distance of 140 mm of the detector surface to the cobalt-60 source. The template values are generated from the average result of 150 measurements on the reference item. Fig 4 shows a gamma spectra acquired with the IBX II of an invalid item compared to that template. A spectrum collapsed into the 12 broad-energy bins is shown in Fig 5, compared to the 12 broad-energy bin values of the template.

**Fig 6:**
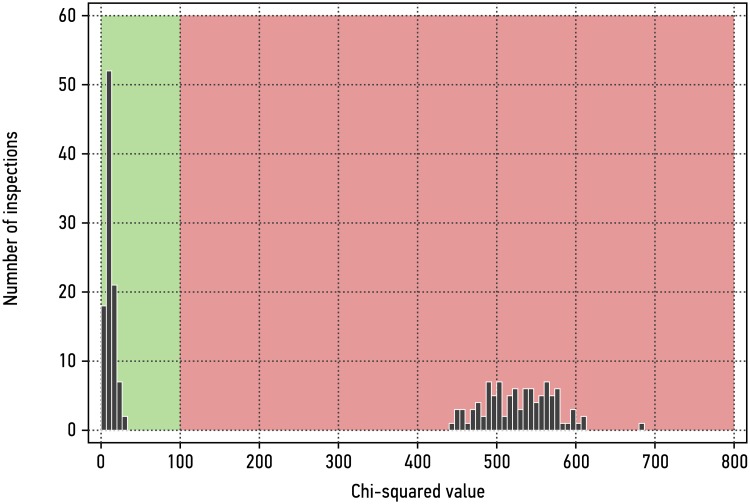
*χ*^2^-result for 150 measurements each on the valid and on the invalid item, compared to the template. *χ*^2^ values for valid and invalid items are clearly distinct. The pass/fail threshold value, here arbitrarily set at $\chi_{\text{T}}^{2}=100$, would have to be negotiated by the parties to meet requirements for false-positive and false-negative rates.

Fig 6 shows *χ*^2^ results for 150 measurements recorded with this routine for valid items (reference items from above) and invalid items compared to the template. Based on the *χ*^2^ test defined in Eq (1), both items can be clearly distinguished by the algorithm.

## Conclusion and future work

In this paper, we set out to develop and demonstrate a prototype of an information barrier (IBX II) that uses vintage hardware that is extremely primitive by today’s standards and may be much less difficult to authenticate than modern electronics. In particular, we have shown that the 6502 processor developed in the early 1970s, which has only 4,237 transistors and typically runs at 1 MHz, is sufficient to process the data acquired with a standard sodium-iodide detector and to execute the statistical tests needed for the template-matching method. Written in machine language, the information barrier software is about 4,000 bytes in size, and execution of the various functions (multichannel analysis, energy calibration, non-linearity correction, re-binning, and *χ*^2^-test) requires less than 1,500 bytes of additional memory. The low-resolution gamma spectra with 256 energy bins, later merged into only 12 broad-energy bins, are sufficient to detect even small differences in the radiation signature with high confidence.

For this proof-of-concept demonstration, we have used an Apple IIe, which is an excellent prototyping platform with a simple and well-understood hardware. Using such a widely available vintage computing platform could be one possible path forward toward a fieldable device that can be simultaneously certified and authenticated so that both the host and the inspector can trust the equipment. Another approach would be to develop an even simpler, single-purpose computer using only the 6502 or another vintage microprocessor and some additional components for RAM and ROM. We are also exploring the possibility of replacing electronic storage media (ROM) by non-electronic ones (punched cards) to increase transparency of the software and to permanently store the data of the template. Finally, we plan to examine methods to confirm the correct design of the 6502 and other hardware components using, for example, high-resolution x-ray microscopy for comparison with the well-known design of the original 6502 circuit. Another way to approach this question would be to directly confirm the age or provenance of the components, which could in turn be a reliable proof of the trustworthiness of the information barrier.

Simplifying information barrier technology to the level demonstrated in this paper may help overcome the lack of trust in hardware and software that has persisted throughout the years and prevented the use of warhead confirmation measurements in an arms-control context. The approach laid out in this paper may therefore offer a new path toward fieldable information barriers, which ideally should be available and ready for use when the next window of opportunity for deeper cuts in the nuclear arsenals opens.
